# Physician reported perception in the treatment of high blood pressure does not correspond to practice

**DOI:** 10.1186/1471-2296-10-23

**Published:** 2009-04-02

**Authors:** Randy Wexler, Terry Elton, Christopher A Taylor, Adam Pleister, David Feldman

**Affiliations:** 1 The Ohio State University, Columbus, Ohio, USA

## Abstract

**Background:**

High blood pressure is a significant health problem world-wide. Physician factors play a significant role in the suboptimal control of hypertension in the United States. We sought to better understand primary care physician's opinions regarding use of hypertension guidelines, patient and physician related barriers to treatment and physician treatment decision making in the management of hypertension as part of a first step in developing research tools and interventions designed to address these issues.

**Methods:**

An IRB approved survey pertaining to physician opinion regarding the treatment of hypertension. Items consisted of questions regarding: 1) knowledge of hypertension treatment guidelines; 2) barriers to hypertension control (physician vs. patient); and 3) self-estimation of physician treatment of hypertension. Descriptive Statistics were used to describe results.

**Results:**

All physicians were board certified in family or general internal medicine (n = 28). Practices were located in urban (n = 12), suburban (n = 14) and inner city locations (n = 1). All physicians felt they did a good job of treating hypertension. Most physicians felt the biggest barrier to hypertension control was patient non-compliance. Half of physicians would fail to intensify treatment for hypertension when blood pressure was above recommended levels for all disease states studied (essential hypertension, heart disease, diabetes, and renal disease).

**Conclusion:**

Physician ability to assess personal performance in the treatment of hypertension and physician opinion that patient noncompliance is the greatest barrier to optimal hypertension control is contradictory to reported practice behavior. Optimal blood pressure control requires increased physician understanding on the evaluation and management of blood pressure. These data provide crucial formative data to enhance the content validity of physician education efforts currently underway to improve the treatment of blood pressure in the primary care setting.

## Background

Hypertension is a significant public health concern affecting more than 1 in 3 Americans [1]. Poorly controlled blood pressure is the common pathway leading to morbidity and mortality in patients with heart disease, diabetes, and renal disease. Reductions in blood pressure (up to by 21–55 mmHg) have been achieved through lifestyle modification, including dietary sodium reduction, the Dietary Approaches to Stop Hypertension eating plan, weight loss, exercise, and moderate alcohol consumption [1-4]. Unfortunately, many patients do not implement such changes for a multitude of reasons; among them include access to healthcare, affordability of medications, and most specifically, lack of education by their physician [5-8].

Physicians often overestimate their effectiveness of the care they provide [9,10]. It has been argued that one possibility for such thinking is a lack of education and training on how to reach therapeutic goals which hinders the ability of physicians to achieve desired treatment targets [11]. Much of the training undertaken by physicians is focused on diagnosis and treatment of symptomatic complaints. As such, medical education may not be adequate to provide the clinician with the structure necessary to provide the desired educational components of treatment: 1) the benefits to treating to therapeutic goals; 2) the complexity of treating to target; and; 3) restructuring practice to facilitate treatment of diseases that can be gauged by symptom relief [11]. The diagnosis of hypertension is the most common diagnosis for primary care physicians in the United States [12]. As such, we sought to better understand primary care physician's opinions regarding use of hypertension guidelines, patient and physician related barriers to treatment and physician treatment decision making in the management of hypertension as part of a first step in developing research tools and interventions designed to address these issues.

## Methods

An Ohio State University Research Foundation Humans Subjects Institutional Review Board approved anonymous survey was conducted during a monthly clinical meeting of family medicine and internal medicine physicians (n = 28). Prior to administering the survey, the purpose of the study was explained in detail, and informed consent was verbally obtained. The survey was administered at the start of the meeting. Physicians completed the survey during the first 10 minutes of the meeting and placed their answers in a large envelope which was then sealed until opened by the research team.

Instrument development was guided by recommendations of Seventh Report of The Joint National Committee on Prevention, Detection, Evaluation, and Management of High Blood Pressure (JNC 7), and research which indentified the sub-optimal control of hypertension in the United States [1,13]. Items were developed to measure physician judgment on degree of blood pressure control based on concomitant disease processes as well as physician opinion on causes of sub-optimal blood pressure control. To improve content validity, specific content of the items was based on previous research as well as JNC 7 guidelines [1,13].

The survey instrument consisted of items pertaining to physician beliefs regarding use of guidelines, barriers to treatment and treatment decision-making in the management of hypertension. Specifically physicians were asked: 1) about their knowledge of hypertension treatment guidelines; 2) self-estimation of their ability to adequately treat hypertension; and 3) barriers to hypertension control (Table 1). Responses to questions were constructed using a standard Likert scale (strongly disagree; disagree, undecided, agree, strongly agree). An additional eight questions asked physicians at which level of blood pressure they would intensify blood pressure treatment for patients with essential hypertension, heart disease, diabetes, and renal disease (Table 2). These questions were based on the recommendations of the Seventh Report of the Joint National Committee on Prevention, Detection, Evaluation, and Management of High Blood Pressure guidelines [1].

**Table 1:** Physician Response to General Questions Regarding Hypertension

**Statement**	**SD**	**D**	**UN**	**A**	**SA**
Control of hypertension is a significant health problem.	0	0	0	5	23
I do a good job of treating my patients' hypertension.	0	0	0	25	3
Clinical practice guidelines are important to follow when treating patients.	0	0	1	15	12
I believe that a significant barrier to blood pressure control is patient non-compliance.	0	0	2	18	8
I believe that a significant barrier to blood pressure control is physician inaction.	0	13	5	9	1
I am familiar with the content of JNC 7	0	1	0	16	11

SD = Strongly Disagree; D = Disagree; UN = Undecided; A = Agree; SA = Strongly Agree

JNC7 = the Seventh Report of the Joint National Committee on the Evaluation, Detection, and Treatment of Hypertension

**Table 2:** Physician Response to Intensification of Hypertension Treatment

**Blood Pressure Estimates**	**MIN**	**MAX**	**MED**	**MEAN**	**SD**
Level of SBP at which to intensify treatment in patients with *essential hypertension*	120	145	140	137.59	5.071
Level of DBP at which to intensify treatment in patients with *essential hypertension*	80	95	90	88.7	3.561
Level of SBP at which to intensify treatment in patients with *heart disease*	120	160	130	134	7.022
Level of DBP at which to intensify treatment in patients with *heart disease*	70	100	85	85.15	5.580
Level of SBP at which to intensify treatment in patients with *diabetes*	120	140	130	130.41	3.320
Level of DBP at which to intensify treatment in patients with *diabetes*	70	90	85	82.93	4.206
Level of SBP at which to intensify treatment in patients with *renal disease*	120	140	130	129.85	4.663
Level of DBP at which to intensify treatment in patients with *renal disease*	70	90	80	82.19	4.216

SBP = Systolic Blood Pressure; DBP = Diastolic Blood Pressure

MIN = Minimum; MAX = Maximum; MED = Median; SD = Standard Deviation

## Results

Seventy-seven per cent of physicians present completed the survey (n = 28). All were board certified in either family medicine or general internal medicine. Sixteen had been in practice ten years or less. Practices were located in urban (n = 12), suburban (n = 14) and inner city location (n = 1). No physicians were in solo practice.

All 28 physicians believed that they do a good job of treating hypertension (25-agree, 3-strongly agree) (Table 1). Twenty-seven physicians felt that the use of guidelines was important (15-agree, 12-strongly agree). Physicians felt that patient non-compliance was the biggest barrier to blood pressure control (18-agree, 8-strongly agree), whereas only 10 (9-agree, 1-strongly agree) felt that physician's factors were the cause.

Questions pertaining to knowledge of the Seventh Report of The Joint National Committee on Prevention, Detection, Evaluation, and Management of High Blood Pressure, found that all but one physician were "familiar" with the guidelines, and all but one felt that they "understood" the guidelines.

Physician responses to questions as to when intensify therapy for a patient with essential hypertension produced a range of 120–145 mmHg systolic (median of 140 mmHg and a S.D. of 5.071), and 80–95 mmHg diastolic (median of 90 mmHg and a S.D. of 3.561) (Table 2). For patients with underlying heart disease, physician response as to when to intensify therapy produced a range of 120–160 mmHg systolic (median 130 mmHg, and a S.D. of 7.022) and 70–100 mmHg diastolic (median 85 with a S.D. of 5.580). With regard to diabetes, physician response as to when to intensify therapy resulted in a range of 120–140 mmHg systolic (median 130 mmHg and a S.D. 3.320), and a range of 70–90 mmHg diastolic (median 85 mmHg with a S.D. of 4.206). When intensifying therapy for a patient with renal disease, physician response resulted in the same range for systolic and diastolic with a median of 130 mmHg systolic (S.D. 4.663) and a median diastolic of 80 mmHg (S.D. 4.216). These data provide crucial formative data to enhance the content validity of physician education efforts currently underway to improve the treatment of blood pressure in the primary care setting.

## Discussion

Physician characteristics may have implications for future blood pressure control. Hyman and Pavlik found that age, sex, specialty, participation in managed care, or perceived usefulness of different types of studies were not significant [13]. However, the one physician characteristic which did reach significance was Board Certification. We were encouraged to find that a significantly larger percentage of physicians we surveyed (96%) were familiar with JNC recommendations than previously reported in the literature (41%) [13]. This may be related to the fact that all of the physicians in our survey were board certified. However, the responses to questions regarding patient and physician contribution to lack of blood pressure control, as well as when to intensify therapy, demonstrate the need for continued educational outreach to physicians who treat patients with hypertension.

In our sample, 36% of respondents felt that physician behavior was a barrier to blood pressure control, while 93% felt that patient non-compliance was a significant barrier. The literature does not support this contention and it has been argued by some physicians that patient non-compliance is 75% physician related [14,15]. In nearly one-third of patient visits, physicians fail to mention blood pressure to patients, and counseling regarding the health impact of blood pressure was discussed even less [8]. It is not realistic to expect patients to comply with treatment when a low level of communication and guidance from physicians exists.

Failure of physicians to intensify therapy in patients with elevated blood pressures also represents a significant area in need of improvement. Although the median level at which physicians would intensify therapy is better than in previously reported research [13], fully 50% still would not intervene at recommended levels even if the patient had concomitant co-morbidities.

Due to the suboptimal control of hypertension in the United States research into interventions designed to improve physician related factors has been performed [16-23]. The success or failure of each method varies from guideline to guideline but some generalities may be drawn. Passive dissemination of information including formal continuing medical education methods has been shown to be ineffective, although interactive workshops may produce some benefit [16,19]. Reminders, either via computer programs or hard copy (paper) tend to improve compliance although the use of computer systems has been demonstrated to have little or no effect [16,19,20]. The use of chart audit and feedback has been shown to be beneficial in some instances, although most find this technique to be equivocal [16,17,19]. Use of local opinion leaders has been shown to produce equivocal results, although the use of academic detailing appears to show some promise [16,19,21-23]. Unfortunately there is no consistent method to date for changing physician behavior and improving compliance with hypertension guidelines. In addressing this issue as it pertains to hypertension specifically, the Agency for Health Care Research and Quality in an extensive review concluded that "the evidence for the use of intervention strategies in hypertension is incomplete and requires further and more detailed evaluation" [18].

Design of interventions for behavioral change, whether physician or patient specific, necessitates an understanding of behavioral theories applicable to the desired goal. Theories that are applicable in the context of our discussion include but are not limited to The Theory of Social Learning (TSL), The Health Belief Model (HBM), and The Theory of Reasoned Action/Planned Behavior.

Albert Bandura's TSL focuses on the theory of perceived self-efficacy [24]. According to Bandura, "Efficacy expectations are a major determinant of people's choices of activities, how much effort they will expend, and of how long they will sustain effort in dealing with stressful situations" [25].

The HBM also addresses perception, but is more expansive than the TSL [26,27]. The HBM has three components: 1) perceived susceptibility (how likely one is to develop a specific illness); 2) perceived seriousness (even if one does have a disease how serious is the outcome likely to be; and 3) perceived benefits (weighing the benefits of an action to the barriers of performing that action). For a person to adopt the desired behavior the individual must consider themselves at high risk for the disease, the disease must have a significant impact on their health, and that action is worth overcoming barriers to action. Though developed to explain patient behavior, it can be applied to understand physician behavior with regards to performing a specific intervention (e.g. improved blood pressure control). The theory has been used to evaluate a variety of health behaviors: 1) disease prevention; 2) health promotion; and 3) treatment compliance [28].

The Theory of Reasoned Action/Planned Behavior states that a person's behavior is determined by his/her intention to perform the behavior and that this intention is formulated based on the individuals concept of normal [29,30]. In other words, intention is the result of the perception of one's usual environment.

This study will inform the content of education interventions by allowing us to develop and test interventions to improve blood pressure control by addressing barriers previously identified in the literature.

Some limitations should be noted. Our population was smaller than those previously studied, and the physicians practice within the same geographic area. However, our intent was to obtain formative data for future work. Additionally, we were specifically interested in teaching faculty as their practice patterns have a greater impact on the future practice of medicine given their work with Residents and Students. As such, we intentionally drew from a physician population who teach students and residents and thus our results may not be generalizable to all primary care practices. However, our results showed a very clear lack of consistency between physician self report of treatment and actual practice. A problem that is not unique to our cohort [13].

## Conclusion

Physician ability to assess personal performance in the treatment of hypertension and physician opinion that patient noncompliance is the greatest barrier to optimal hypertension control is in conflict with reported practice behavior. Given the time constraints of today's primary care physician work force and the continued lack of optimal blood pressure control, focused educational messages, and simplified treatment algorithms should be investigated for use in the primary care setting. These data provide crucial formative data to enhance the content validity of physician education efforts currently underway to improve the treatment of blood pressure in the primary care setting.

## Competing interests

Wexler: Receives research funding from Pfizer, and CVRx. Speaker for CVRx. DSMB for Cardiomems Inc. Elton: Receives research funding from the NIH. Feldman: Receives research funding from NIH, and Medtronic. Consultant for GSK. Speakers Bureau for GSK, Novartis, JNJ

## Authors' contributions

RW conceived of the study, and participated in its design, coordination, and analysis, and helped draft the manuscript. TE reviewed the data and helped draft the manuscript. CT reviewed and analyzed the data and help draft the manuscript. AP reviewed the data and helped draft the manuscript. DF conceived of the study, and participated in its design, coordination, and analysis, and helped draft the manuscript. All authors read and approved the final manuscript.

## Authors' information

Randy Wexler MD, MPH, FAAFP is an assistant professor of Clinical Family Medicine at the Ohio State University College of Medicine.

Terry Elton, PhD, is Professor in the Davis Heart and Lung Research Institute and College of Pharmacy, Division of Pharmacology, at The Ohio State University.

Christopher Taylor PhD, RD, LD is an Assistant Professor of Health, Wellness and Medical Dietetics at the Ohio State University College of Medicine.

Adam Pleister, MD, is a Fellow in the Division of Cardiovascular Medicine – Department of Internal Medicine at The Ohio State University College of Medicine

David Feldman, MD, PhD, FACC is an associate professor of Medicine and Cardiology, and physiology and cell biology at the Ohio State University College of Medicine.

## Pre-publication history

The pre-publication history for this paper can be accessed here:


